# Multimorbidity and its associations with physical function, fall risk, and hospitalization cost in adults with type 1 diabetes: a cross-sectional study

**DOI:** 10.3389/fendo.2026.1817819

**Published:** 2026-05-07

**Authors:** Minxi Lao, Xiang Zhang, Xiaoyi Ma, Jingya Wang, Jieming Mai, Haipeng Xiao, Jin Li, Lei Su

**Affiliations:** 1 Department of Geriatrics, First Affiliated Hospital of Sun Yat-Sen University, Guangzhou, China; 2 School of Health Sciences, College of Medicine and Health, University of Birmingham, Birmingham, United Kingdom; 3 Department of Endocrinology, First Affiliated Hospital of Sun Yat-Sen University, Guangzhou, China

**Keywords:** activity of daily living, comorbidity burden, fall risk, hospitalization cost, type 1 diabetes

## Abstract

**Background:**

Multiple long-term conditions co-occur in people with type 1 diabetes. We aim to investigate the association between comorbidities and physical function, fall risk and hospitalization cost.

**Methods:**

A cross-sectional study was conducted at the First Affiliated Hospital of Sun Yat-sen University. Adult patients with type 1 diabetes admitted between 2021 and 2025 were included. Prevalence of each morbidity was compared in people with different diabetes duration. ADL was assessed by the Barthel Index. Fall risk was evaluated by the Johns Hopkins Fall Risk Assessment Tool. Logistic regression models were used to analyze the association between multimorbidity and physical function and fall risk. Linear regression was used between eight variables and hospitalization costs. Variables associated with increasing risk of multimorbidity were identified using multivariate logistic regression model.

**Results:**

The mean number of morbidities per patient was 3.6 ± 1.8, with 31.4% (n=118), 41.5% (n=156), and 27.1% (n=102) had 1-2, 3-4, and ≥5 morbidities. Cardiovascular, kidney, metabolic conditions were the most prevalent comorbidities. Cataract, anemia, cancer, autoimmune thyroid disorders, chronic obstructive pulmonary disease, and mental health disorders were also notable. Older age and longer diabetes duration were strongly associated with higher multimorbidity burden. Increased multimorbidity was associated with higher fall risk (odds ratio (OR): 1.23, 95% confidence interval (CI): 1.01-1.52) (P<0.05), greater dependence in Activities of daily living (OR: 1.58, 95% CI: 1.08-2.32) (P = 0.02) and elevated hospitalization costs (β: 66.12, 95% CI: 14.65–117.58) (P = 0.012).

**Conclusions:**

Our findings demonstrate that multimorbidity is highly prevalent among Chinese adults with T1D. A higher burden of multimorbidity is significantly associated with adverse functional outcomes, including increased fall risk and greater dependence in ADL, as well as higher hospitalization costs. These findings highlight the critical need to integrate assessments of functional status, fall risk, and multimorbidity into routine clinical care for adults with T1D.

## Introduction

Life expectancy for individuals with type 1 diabetes (T1D) has increased significantly (1). As a result, the prevalence of T1D among older adults has been rising steadily (2). Multimorbidity, the coexistence of two or more chronic medical conditions in an individual, is a growing global health challenge (3). Therefore, understanding the complex interplay between T1D and multimorbidity is essential in clinical practice and research.

T1D was considered to co-occurred with certain autoimmune diseases (e.g. Hashimoto thyroiditis, Graves’ disease, celiac disease, autoimmune hepatitis, myasthenia gravis) previously (4). However, observational studies reveal that adult patients with T1D are often multimorbid. An electronic health record-based study on 6967 German outpatients found each T1D individual is diagnosed with 3.1 different disorders. The most common conditions included hypertension (31.2%), dyslipidemia (26.4%), dorsalgia (20.4%), neuropathy (17.3%), and depression (14.6%) (5). A meta-analysis also showed a high prevalence of mental disorders (e.g., depressive, anxiety, and eating disorders) in patients with T1D (6). These findings broaden our understanding of multimorbidities associated with T1D.

However, the impact of multimorbidities on T1D patients remains unclear. An association study using health insurer database found that individuals with T1D who had autoimmune diseases are at higher risk of renal failure, ischemic stroke, and myocardial infarction (4). A population-based study found each additional morbidity at the age of 50 years is associated with four years of life shorter in people with diabetes (7). However, the data was not able to differentiate T1D from type 2 diabetes (T2D).

Until now, it is unclear whether multimorbidity is associated with reduced functional status and increased hospitalization cost in T1D. Activities of daily livings (ADL) is considered as a patient-centered outcome that reflects a person’s ability to perform essential self-care tasks. This functional status is a stronger predictor of morbidity and mortality than many other physiological or laboratory measures (8). Older adults with diabetes are at risk for impairments in ADL performance (9). Fall risk is a common and serious safety issue for patients with diabetes. Older adults with diabetes are 1.5 times more likely to fall than those without diabetes (10). Hospitalization cost shows the economic burden of multimorbidity (11). In this study, we aim to assess: (1) The prevalence of multimorbidity in individuals with type 1 diabetes in our hospital. (2) Impact of multimorbidity on three adverse outcomes (ADL dependence, fall risk and hospitalization cost).

## Methods

### Study design and population

This cross-sectional study was conducted in the First Affiliated Hospital of Sun Yat-sen University. The study was approved by the Research Ethics Committee of the First Affiliated Hospital of Sun Yat-sen University. Individual consent was waived. The study was performed in accordance with the Declaration of Helsinki. No patients or members of the public were directly involved in the design, conduct, or dissemination of this research.

T1D patients admitted to the hospital between January 2021 and June 2025 were initially identified using International Classification of Diseases-10 (ICD-10) codes and re-evaluated by two clinicians independently. The reasons for patient admission include the following: newly diagnosed diabetes; poorly controlled blood glucose requiring intensive insulin therapy; concurrent with stress conditions such as high fever, severe respiratory infections, acute cholecystitis, urinary tract infections, acute cardiovascular and cerebrovascular diseases; severe chronic complications such as diabetic nephropathy, diabetic fundus hemorrhage, painful neuropathy, intractable diarrhea, and foot gangrene; and acute diabetic complications such as diabetic ketoacidosis, lactic acidosis, and frequent hypoglycemia. A total of 376 adult patients (age >18 years) were included in the final analysis (consort diagram in **Supplementary Figure 1**).

### Multimorbidity definition and ascertainment

The definition of multimorbidity is the simultaneous occurrence of two or more chronic conditions in the same individual, regardless of cause (including T1D). Moderate multimorbidity was defined as the presence of 3–5 morbidities. Severe multimorbidity was defined as ≥5 morbidities. Comorbidity was defined as the existence of one or more distinct additional health conditions in a patient who already has an index disease (T1D).

All conditions were identified by ICD-10 codes, and key diabetes-related complications used combined clinical criteria + ICD-10 coding for accuracy. T1D was diagnosed according to the glucose criteria established by the American Diabetes Association (12). Latent autoimmune diabetes in adults (LADA) is also classified under the diagnosis of T1D. Islet autoantibodies (Glutamic acid decarboxylase, islet tyrosine phosphatase 2, zinc transporter 8) were measured as surrogate markers of pancreatic β-cell destruction. C-peptide was measured to indicated low insulin secretion. Demographic information was collected by the physicians in charge of the patients, including age, gender, body mass index [BMI = weight (kg)/height (m)²], and disease duration.

A diagnosis of dyslipidemia was made when one or more of the following fasting serum lipid levels are met: Total Cholesterol (TC): ≥ 6.2 mmol/L (≥ 240 mg/dL) or Low-Density Lipoprotein Cholesterol (LDL-C): ≥ 4.1 mmol/L (≥ 160 mg/dL) or Triglycerides (TG): ≥ 2.3 mmol/L (≥ 200 mg/dL). Hypertension was defined as systolic blood pressure ≥140 mmHg or diastolic blood pressure ≥90 mmHg for adults, and/or a history of antihypertensive therapy. Diabetic retinopathy, cataract and glaucoma was assessed by ophthalmologists using retinal photography and tonometer. Diabetic kidney disease was defined by the estimated glomerular filtration rate, the presence of albuminuria, or the hemodialysis stage. Diabetic peripheral neuropathy was established upon the presence of symptoms and/or signs of peripheral nerve dysfunction, after the exclusion of other potential causes. It typically required a combination of the following: neuropathic symptoms (e.g., numbness, tingling, pain, or weakness in a distal stocking-glove distribution), decreased distal sensation upon sensory examination (e.g., to pinprick, light touch, vibration, or temperature), and diminished or absent ankle reflexes. Nerve conduction velocity studies were also performed in a subset of patients. Peripheral vascular disease was defined as the symptomatic atherosclerotic disease and ultrasound suggests atherosclerotic plaques in the lower extremity arteries. Obesity is diagnosed with BMI >28 kg/m^2^. Nonalcoholic fatty liver disease was diagnosed with ultrasonography after investigation for other causes of elevated liver enzymes. The diagnosis of Hashimoto’s thyroiditis was confirmed by elevated levels of thyroid peroxidase antibodies (TPO antibodies), often accompanied by hypothyroidism on thyroid function tests. For hypothyroidism, we excluded those with postsurgical, postablative, iodine, or iatrogenic hypothyroidism. Transient anemia has been ruled out. All diagnoses at discharge were coded based on the ICD-10 by certified coders in our hospital. A total of 61 diagnosed chronic physical and mental diseases were included as multimorbidities, in accordance with the literature and expert consensus (**Supplementary Table 2**) (7, 13, 14).

### Outcome measures

#### Activities of daily living

The Barthel Index (ADL) assesses mobility, transfer, ambulation, and stair climbing—core components of physical function widely used in hospitalized adult populations. The index evaluates ten items: feeding, bathing, grooming, dressing, bowel control, bladder control, toileting, chair-bed transfer, ambulation on a level surface (45 meters), and stair climbing. The total score ranges from 0 to 100, with higher scores indicating greater independence. ADL is categorized as follows: 61–99 for mild dependence; 41–60 for moderate dependence; 21–40 for severe dependence; and ≤20 for complete dependence (15). ADL was assessed by the nurse in charge when the patient was admitted and was re-assessed based on their risk levels and changes in medical conditions during the hospital stay. The most recent pre-discharge assessment was selected for analysis due to the patient’s stable condition at that time.

#### Fall risk

The Johns Hopkins Fall Risk Assessment Tool was used to evaluate fall risk (16), with a total score of 35. Higher scores indicate a greater fall risk: ≤5 suggests low risk, 6–13 suggests moderate risk, and >13 suggests high risk (17). The fall risk score was assessed by the responsible nurses. The assessment results were extracted from the medical record.

#### Hospitalization cost

The hospitalization cost per admission represents the pre-insurance total during that particular hospital stay, covering diagnostics, lab tests, nursing, and medications. All values are converted to USD using the RMB/USD exchange rate as of August 15, 2025.

### Statistical analyzes

Continuous variables that followed a normal distribution were expressed as mean ± SD. Continuous variables that did not follow normal distribution were expressed as median (interquartile range [IQR]). Categorical variables (e.g., gender, ADL dependence, fall risk, degree of obesity, HbA1c) were presented as numbers (percentages). For comparisons among three groups, one-way ANOVA was used for normally distributed data, and the Kruskal–Wallis test was applied for non-normally distributed data. Prevalence of multimorbidities in those with T1D was calculated. The numerator was the number of patients with T1D who had evidence of the disease diagnosis from the records. The denominator was the number of patients with T1D. All diagnoses were derived from inpatient discharge records during the index admission, eliminating misclassification.

Logistic regression models adjusted for age, sex, disease duration, BMI, HBA1c were used to estimate the association between multimorbidity and ADL dependence and fall risk. Linear regression was used between eight variables (ADL, fall risk, disease course, BMI, HbA1c, age, length of hospital stay, and number of multimorbidities) and hospitalization costs. The number of morbidities was treated as a continuous variable in multivariate logistic regression (for ADL dependence and fall risk) and multivariate linear regression (for hospitalization cost), to estimate the effect of each additional morbidity. The number of morbidities was categorized into 1–2, 3–4, and ≥5 groups for descriptive analysis and prevalence presentation only.

P values <0.05 were considered statistically significant. All statistical analyzes were performed using SPSS 24.0.and R version 4.4.2.

## Results

### Clinical characteristics

A total of 376 patients with T1D were included. The mean age (standard deviation [SD]) was 46.22 (16.73) years. The median (IQR) disease duration of T1D was 60 (12,156) months. Clinical characteristics are shown in **Table 1**. Each patient was diagnosed with 3.6 ± 1.8 morbidities on average (including T1D). Among the 376 patients, 31.4% (n=118), 41.5% (n=156), and 27.1% (n=102) had 1-2, 3-4, and ≥5 morbidities, respectively. Older patients had a longer disease course, more morbidities, and higher hospitalization costs compared with younger groups, when stratified by three age groups: 18–39 years (n=152), 40–59 years (n=123), and ≥60 years (n=101). 18–39, 40–59, ≥60 years represent young, middle-aged, and older adults with T1D.

**Table 1:** Baseline characteristics of study population.

Variables	Total (n = 376)	Age, years			*P*
		18-39 (n = 152)	40-59 (n = 123)	>=60 (n = 101)	
Age, years, mean ± SD	46.22 ± 16.73	28.99 ± 6.60	50.62 ± 5.70	66.8 ± 6.4	<0.01
Disease duration, months, median (IQR)	60.00 (12.00, 156.00)	42.00 (6.00,120.00)	72.00 (12.00,144.00)	120.00 (12.00,240.00)	<0.01
Number of multimorbidities, mean ± SD	3.61 ± 1.77	2.73 ± 1.43	3.77 ± 1.63	4.72 ± 1.71	<0.01
Cost, US$, median, (IQR)	1154.71 (915.04, 1609.09)	1042.40 (839.75,1558.68)	1171.01 (929.60,1527.44)	1257.59 (1016.23,1821.86)	<0.01
Gender, n (%)					0.54
Male	186 (49.47)	70 (46.05)	63 (51.22)	53 (52.48)	
ADL dependence, n (%)					0.64
Mild	365 (97.07)	148 (97.37)	120 (97.56)	97 (96.04)	
Moderate	6 (1.60)	3 (1.97)	1 (0.81)	2 (1.98)	
Severe	3 (0.80)	0 (0.00)	2 (1.63)	1 (0.99)	
Complete	2 (0.53)	1 (0.66)	0 (0.00)	1 (0.99)	
Degree of fall risk, n (%)					<0.01
Low	335 (89.10)	142 (93.42)	114 (92.68)	79 (78.22)	
Moderate	36 (9.57)	9 (5.92)	6 (4.88)	21 (20.79)	
High	5 (1.33)	1 (0.66)	3 (2.44)	1 (0.99)	
BMI, kg/m^2^, n (%)					<0.01
<18.5 (underweight)	85 (22.73)	47 (31.13)	23 (18.85)	15 (14.85)	
18.5-24 (normal)	192 (51.34)	73 (48.34)	59 (48.36)	60 (59.41)	
≥24 (overweight)	97 (25.94)	31 (20.53)	40 (32.79)	26 (25.74)	
HbA1c, %, n (%)					0.01
<6.5	50 (13.30)	23 (15.13)	21 (17.07)	6 (5.94)	
6.5-9	154 (40.96)	64 (42.11)	38 (30.89)	52 (51.49)	
>=9	172 (45.74)	65 (42.76)	64 (52.03)	43 (42.57)	

ADL, activity of daily living; HbA1c, hemoglobin A1c; BMI, body mass index; SD, standard deviation; IQR, interquartile range.

### Number of multimorbidities among patients of different gender and age

**Figure 1:**
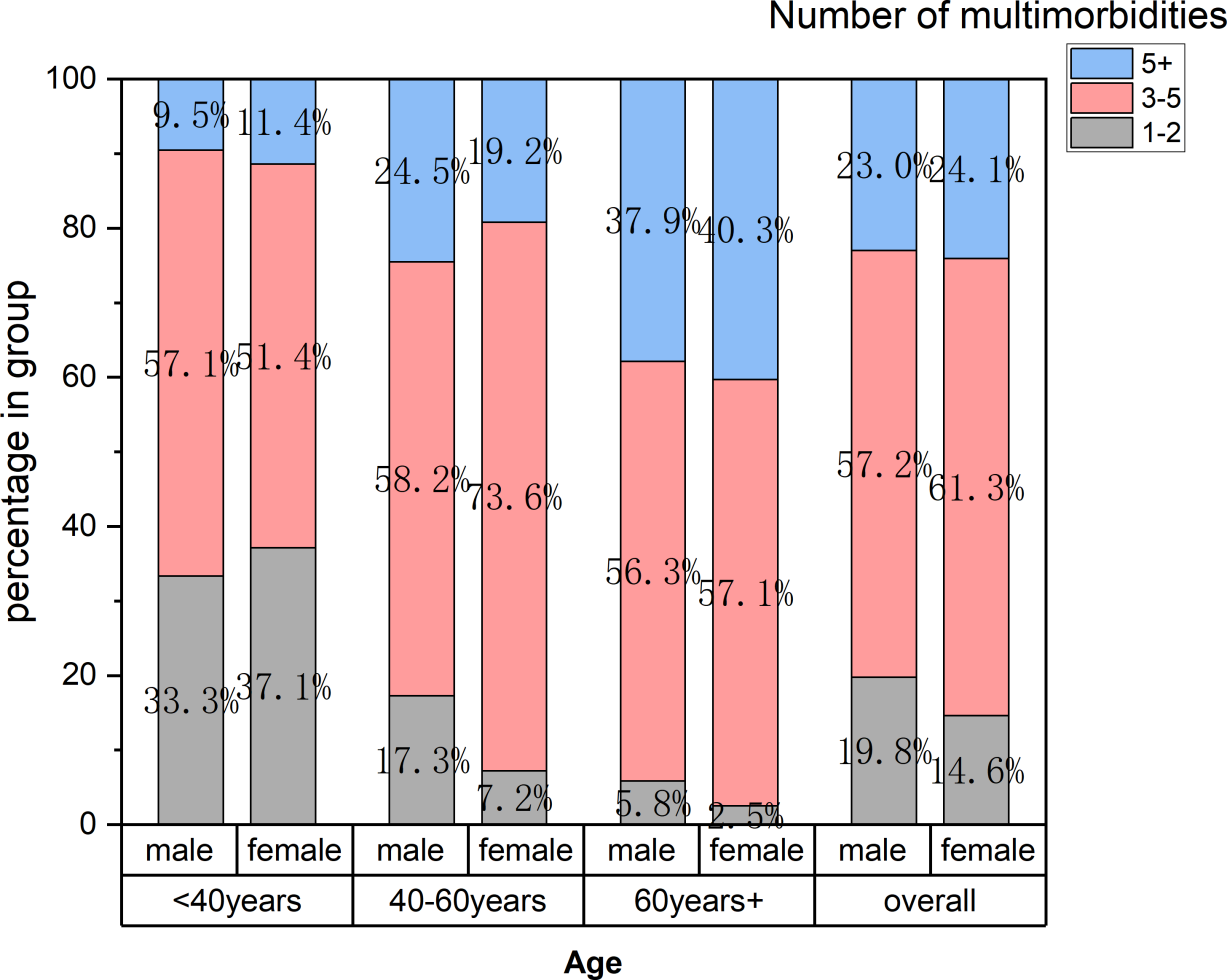
The proportion of patients with type 1 diabetes who had 1-2, 3–5 or ≥5 multimorbidities by age and gender group.

The number and prevalence of these disorders per patient were calculated and stratified by age group (**Figure 1**). Overall, moderate multimorbidity was present in 57.2% in males and 61.3% in females. Severe multimorbidity was present in 23.0% in male and 24.1% in female.

In the 18–39 years group, the majority of patients had 3–5 morbidities (57.1%), while a smaller proportion had 1-2 (33.3% in male, 37.1% in female) or 5+ morbidities (9.5% in male and 11.4% in female). Within the 40–59 years age group, the proportion of individuals with 3–5 morbidities increased to 73.6% (female), and those with ≥5 comorbidities constituted 24.5% in male and 19.2% in female. The oldest group (≥60 years) carried the heaviest disease burden, with 37.9% of male and 40.3% of female patients having more than 5 morbidities. No significant difference was found in the number of comorbidities between gender.

### Distribution of multimorbidities in patients with different disease duration

**Figure 2:**
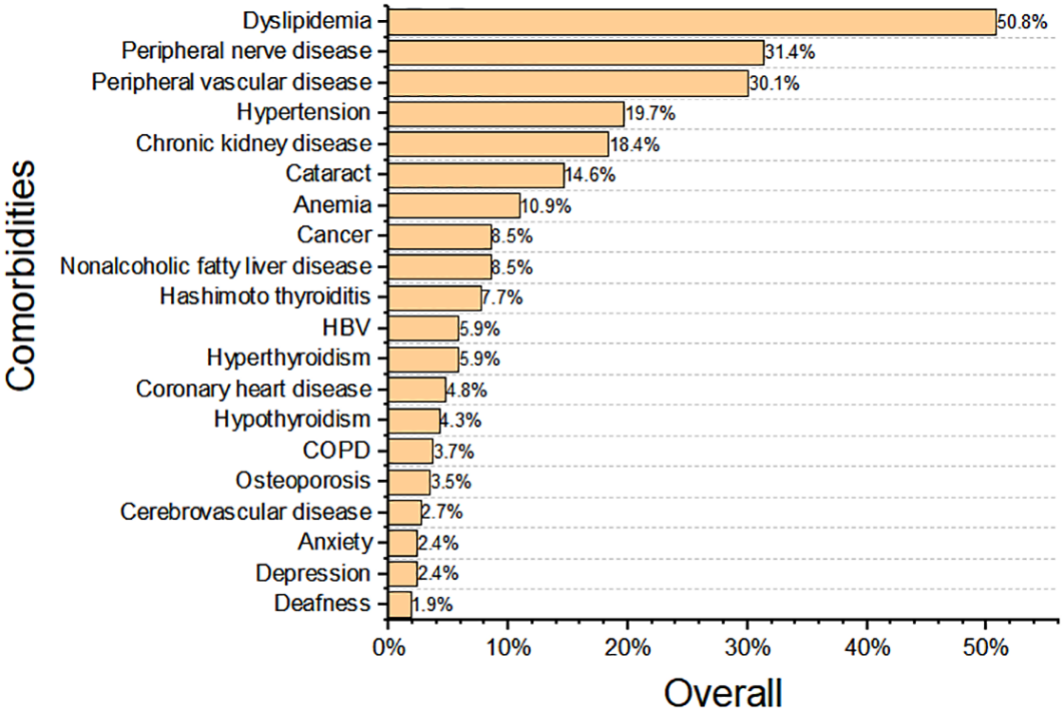
Prevalence of comorbid conditions among adults with type 1 diabetes (top 20). HBV, Chronic Type B Hepatitis; COPD, Chronic obstructive pulmonary disease.

We investigated the prevalence of multiple long-term conditions in adults with T1D (**Figure 2**). Dyslipidemia emerges as the most prevalent comorbidity, affecting 50.8% of the T1D patients, followed by peripheral nerve disease (31.4%), peripheral vascular disease (30.1%), hypertension (19.7%), chronic kidney disease (18.4%). Other relatively common comorbidities in this cohort include cataract (14.6%), anemia (10.9%), and cancer (8.5%). T1D and autoimmune thyroid disease occurred together frequently. The prevalences of Hashimoto’s thyroiditis, hyperthyroidism, and hypothyroidism were 7.7%, 5.9%, and 4.3%, respectively. Coronary heart disease (4.8%), chronic obstructive pulmonary disease (COPD) (3.7%), osteoporosis (3.5%), cerebrovascular disease (2.7%) exhibited relatively low prevalence (<5%) in this cohort. Mental health disorders (e.g., depression (2.4%) and anxiety (2.4%)), also appear within the top twenty.

We investigated the prevalence of comorbid conditions stratified by different disease duration (<5 years, 5–10 years, ≥10 years) (**Supplementary Figure 2**). Conditions such as retinopathy, neuropathy, and chronic kidney disease showed a positive gradient with longer diabetes duration. For example, the prevalence of peripheral nerve disease increased from 19.8% in patients with short disease duration (0–4 years) to 44.6% in patients with long disease duration (≥10 years). The prevalence of chronic kidney disease increased from 6.4% in patients with short disease course, to 34.5% in those with long disease course. A similar trend was observed for hypertension, coronary heart disease, cataract and anemia. In contrast, the prevalence of autoimmune thyroid disease, COPD and osteoporosis remained stable across diabetes durations. These diverse trajectories emphasize the importance of disaggregating comorbidity patterns by diabetes duration when planning surveillance programs.

### Multimorbidity was associated with activities of daily living dependence

We evaluated the associations between number of multimorbidities, other covariates (age, gender, BMI, HbA1c, and disease duration) and ADL dependence, using both univariable and multivariable logistic regression models (**Table 2**). The number of multimorbidities was significantly associated with ADL dependence in the multivariable model (OR = 1.58, 95% CI: 1.08–2.32, *p* = 0.02). Although both normal weight (OR = 0.25,95% CI:0.06 ~ 1.03, *p* = 0.05) and overweight (OR = 0.11, 95% CI:0.01 ~ 1.25, *p* = 0.08) groups showed a trend toward reduced risk compared to the underweight reference group, these associations did not reach statistical significance in multivariable model. Other variables, including age, gender, HbA1c, and disease course were not associated with ADL dependence (all p>0.05), indicating these factors did not independently predict functional dependence.

**Table 2:** Associations between six variables (age, gender, disease course, BMI, HbA1c, and number of multimorbidities) and activities of daily living dependence.

Variables	Univariable logistic regression analysis		Multivariable logistic regression analysis	
	OR (95%CI)	*P*	OR (95%CI)	*P*
Age, years				
<40	1.00 (Reference)	\	1.00 (Reference)	\
40-60	0.93 (0.20 ~ 4.24)	0.92	0.76 (0.14 ~ 4.11)	0.75
>=60	1.54 (0.38 ~ 6.28)	0.55	1.31 (0.22 ~ 7.82)	0.77
Gender				
male	1.00 (Reference)	\	1.00 (Reference)	\
female	1.83 (0.53 ~ 6.35)	0.34	1.51 (0.41 ~ 5.55)	0.54
Disease duration, years				
<5	1.00 (Reference)	\	1.00 (Reference)	\
5-10	1.35 (0.33 ~ 5.58)	0.68	1.15 (0.25 ~ 5.26)	0.86
>=10	0.40 (0.08 ~ 2.02)	0.27	0.30 (0.05 ~ 1.82)	0.19
BMI kg/m^2^				
Underweight	1.00 (Reference)	\	1.00 (Reference)	\
Normal	0.28 (0.08 ~ 1.03)	0.06	0.25 (0.06 ~ 1.03)	0.05
Overweight	0.14 (0.02 ~ 1.18)	0.07	0.11 (0.01 ~ 1.25)	0.08
HbA1c (%)				
<6.5	1.00 (Reference)	\	1.00 (Reference)	\
6.5-9	0.20 (0.03 ~ 1.25)	0.07	0.15 (0.02 ~ 1.05)	0.06
>=9	0.56 (0.13 ~ 2.31)	0.42	0.33 (0.07 ~ 1.60)	0.17
Number of multimorbidities	1.31 (0.97 ~ 1.77)	0.07	1.58 (1.08 ~ 2.32)	0.02

BMI, body mass index; HbA1c, hemoglobin A1c.

### Multimorbidity was associated with fall risk

We explored the associations between number of multimorbidities, other covariates (including age, gender, disease course, BMI, and HbA1c) and fall risk, using both univariable and multivariable logistic regression models (**Table 3**). The number of multimorbidities was associated with fall risk (OR = 1.23, 95% CI: 1.01-1.52, *p* < 0.05) (**Table 3**). As expected, age exhibited a clear and statistically significant association with fall risk (OR (95% CI): 3.87 (1.75-8.56), *p* < 0.01), particularly in the older adults (>60 years). In contrast, gender, disease course, BMI, and HbA1c do not show significant associations with fall risk.

**Table 3:** Associations between six variables (age, gender, disease course, BMI, HbA1c, and number of multimorbidities) and fall risk.

Variables	Univariable		Multivariable model	
	OR (95%CI)	*P*	OR (95%CI)	*P*
Age, years				
<40	1.00 (Reference)	\	1.00 (Reference)	\
40-60	1.14 (0.45 ~ 2.90)	0.78	1.09 (0.40 ~ 2.96)	0.87
>=60	3.87 (1.75 ~ 8.56)	<.01	2.94 (1.15 ~ 7.53)	<0.05
Gender				
male	1.00 (Reference)	\	1.00 (Reference)	\
female	1.33 (0.70 ~ 2.56)	0.39	1.13 (0.57 ~ 2.25)	0.72
Disease course, years				
<5	1.00 (Reference)	\	1.00 (Reference)	\
5-10	0.90 (0.31 ~ 2.58)	0.84	0.92 (0.31 ~ 2.73)	0.87
>=10	1.86 (0.92 ~ 3.76)	0.08	1.62 (0.73 ~ 3.59)	0.24
BMI kg/m^2^				
<18.5(underweight)	1.00 (Reference)	\	1.00 (Reference)	\
18.5-24(normal)	0.83 (0.38 ~ 1.81)	0.64	0.59 (0.25 ~ 1.38)	0.22
>=24(overweight)	0.61 (0.23 ~ 1.59)	0.31	0.35 (0.12 ~ 1.03)	0.06
HbA1c				
<6.5	1.00 (Reference)	\	1.00 (Reference)	\
6.5-9	1.11 (0.39 ~ 3.19)	0.84	0.73 (0.24 ~ 2.23)	0.58
>=9	1.10 (0.39 ~ 3.11)	0.86	0.87 (0.29 ~ 2.61)	0.81
Number of multimorbidities	1.32 (1.12 ~ 1.56)	<0.01	1.23 (1.01 ~ 1.52)	<0.05

BMI, body mass index; HbA1c, hemoglobin A1c.

### Correlation between multimorbidities and hospitalization cost

We investigated the linear relationships between eight critical variables (ADL, fall risk, disease duration, BMI, age, HbA1c, length of hospital stay, and number of multimorbidities) and hospitalization costs (**Supplementary Figure 3**).

The adjusted multivariate linear regression model revealed that the number of multimorbidities was an independent predictor of higher hospitalization cost (β=66.12, 95%CI:14.65–117.58, p =0.012), even after adjusting for length of stay and other covariates. As expected, length of hospital stay was a strong determinant of cost (β=169.78, 95%CI:147.16–192.39, p <0.001). Compared to patients with mild ADL dependence, moderate (β=835.23, 95%CI:210.78–1459.69, p =0.009), severe (β=2817.26, 95%CI:1950.41–3684.10, p <0.001), and complete (β=1632.43, 95%CI:587.55–2677.31, p =0.002) ADL dependence were all associated with significantly higher hospitalization costs. No significant associations were observed for fall risk, diabetes duration, BMI, HbA1c level, or age with hospitalization cost in the adjusted model.

### Factors associated with the number of multimorbidities

We identified key factors associated with increased risk of having multimorbidities among the study population (**Supplementary Table 1**). Age was strongly associated with increased multimorbidity risk. People aged 40–60 had significantly higher risk (OR ranged from 2.26 to 4.45, all *p* < 0.05) compared to the 18–39 reference group. Those ≥60 years old exhibited even greater risk (OR ranged from 6.76 to 10.8, all *p* < 0.05). Disease duration ≥10 years (vs. 0–4 years) increased the risk of multimorbidity (OR range: 2.26 to 3.32, all *p* < 0.05). Gender, BMI, and HbA1c showed no significant association with multimorbidity in any model.

## Discussion

This study provides a comprehensive analysis of multimorbidities and the functional and economic impact on adults with type 1 diabetes (T1D). Our findings reveal several important patterns and offer clinically relevant insights. We found a high burden of multimorbidities among Chinese adults with T1D. A higher number of multimorbidities was associated with increased fall risk, greater dependence on activities of daily living, and substantially higher hospitalization costs.

First, the high prevalence of multimorbidity (80.2% in male and 85.4% in female with ≥3 morbidities) underscores the complex clinical profile of adults with T1D. The prevalence of multimorbidity is higher in patients with T1D than in the general population, where a cohort study of 512,723 Chinese adults aged 30–79 found a rate of 15.8% (18). The China Kadoorie Biobank mainly recruited a community-based population cohort, in which disease status was self-reported, and diabetes was not differentiated between type 1 and type 2. In contrast, our study enrolled hospitalized patients with type 1 diabetes whose diagnoses were made by physicians, supported by auxiliary examinations and laboratory tests, thus achieving a finer granularity of disease diagnosis.

In our study, the most common comorbidities, e.g., dyslipidemia, peripheral vascular disease, neuropathy, hypertension, and chronic kidney disease—reflect the prominent cardiovascular-kidney-metabolic burden in this population. This pattern aligns with previous studies highlighting the accelerated development of metabolic complications in T1D (19, 20) (21). Population-based studies have shown that the most common comorbidities in people with T2D are hypertension, coronary heart disease, asthma, chronic kidney disease, hypothyroidism, atrial fibrillation, COPD, stroke, and rheumatic arthritis (22). The co-occurrence of these conditions implies shared underlying pathophysiological mechanisms—such as chronic hyperglycemia, insulin resistance, systemic inflammation, and oxidative stress—that lead to end-organ damage over time. We also observed that patients with different durations of diabetes exhibited distinct comorbidity profiles. These differences highlight the importance of developing duration-specific screening strategies to mitigate complications. A recent prospective study further demonstrated that the sequence of disease onset—specifically, initial diabetes diagnosis followed by psychosis and then congestive heart failure—is associated with reduced life expectancy compared to other sequences involving the same three conditions (23). Several factors may explain the relatively lower prevalence of mental disorders observed in our study compared to previous reports. First, our study primarily utilized routine clinical diagnoses documented in electronic health records. This approach may under-identify individuals with mild or undiagnosed symptoms who do not seek formal clinical care. Second, cultural attitudes toward mental health and the availability of mental health services across regions can influence prevalence rates.

We also identified the risk factors for multimorbidity in people with T1D. Older age and longer disease duration likely associated with multimorbidity through cumulative metabolic and inflammatory pathways. Overweight exacerbates this process by amplifying insulin resistance and chronic inflammation. These mechanisms interact synergistically, driving both comorbidity accumulation and functional decline in T1D. Interestingly, HbA1c was not significantly associated with multimorbidity burden in most models in our population. Our results suggest that while hyperglycemia is important in diabetes-specific complication, other factors such as genetic predisposition, lifestyle, healthcare access, and inflammatory factors may also play substantial roles in multimorbidity development (24). A 50+ year-old cohort study of 25,931 older adults found that behavioral risk factors—including high BMI, smoking, and low physical activity—were associated with 15 self-reported chronic conditions (25). A systematic review from eight longitudinal studies identifies that in low- and middle-income countries, older age, female gender, lower socioeconomic status, and unhealthy behaviors like smoking are key determinants for developing multimorbidity (26). These findings support a broader approach to risk reduction that extends beyond glycemic control alone.

A novel contribution of this study is the demonstration of functional consequences of multimorbidity. Previous meta-analysis showed that ≥2 comorbidities were associated with hospitalization and with readmission (27). Multimorbidity-derived clusters had higher risk for all-cause mortality, hospitalizations, and general practice use (28). Our findings revealed that multimorbidity is associated with three adverse outcomes, which serves as an important complement to previous results. Falls and functional decline are critical geriatric syndromes that significantly influence quality of life, healthcare utilization, and mortality (29). The incidence of falls requiring hospital treatment was 13.3% in T1D in a cohort study (30). That this association is evident in a T1D population, including those at middle age, suggesting early onset of functional aging. This highlights the need for regular assessment of functional status and fall risk in routine diabetes care.

This study further found that multimorbidity was associated with a significant increase in hospitalization cost per admission. It indicates that multimorbidity elevates resource intensity, typically necessitating longer hospital stays, more frequent consultations, and more complex medications. This aligns with health economic studies across different chronic diseases showing that multimorbidity increases healthcare expenditures substantially (31). The combination of functional decline and increased healthcare cost creates a dual burden on patients and healthcare systems. Our findings strongly argue for promoting integrated, preventive, and patient-centered care models in the long-term care of type 1 diabetes.

The study’s strengths include a clearly defined T1D cohort with rigorous diagnostic confirmation via autoantibodies and C-peptide and use of validated tools (Barthel Index for ADL, Johns Hopkins Fall Risk Assessment). The high multimorbidity prevalence and associations with ADL dependence (OR 1.58), fall risk (OR 1.23), and costs (β ≈101–106 USD per additional morbidity) are novel for T1D-specific inpatients and extend beyond prior outpatient-focused studies (5). Stratification by duration and age adds clinical utility, highlighting duration-dependent rises in neuropathy/CKD.

Several limitations should be considered. The cross-sectional design prevents establishment of causality. Prospective data are needed to determine whether early intensive multifactorial intervention can reduce the incidence of diabetes complications. Residual confounding by unmeasured factors such as socioeconomic status, lifestyle behaviors, and healthcare access is possible. We only included the number of multimorbidities and did not account for the weight of each individual condition or the interactions between them (32). Cause of admission was not included in the cost model, which may be residual confounders. Future studies should adjust for these variables. Due to the limited number of patients with T1D from a single center, future research should involve multi-center collaborations and more detailed analysis of each comorbidity. Finally, the generalizability of our findings to the broader T1D population may be limited, as the study involved an inpatient cohort with likely greater disease severity. ADL/fall assessments are nurse-reported and pre-discharge (potentially biased by acute illness resolution).

Despite these limitations, our findings have important implications for clinical practice and research. They support the implementation of comprehensive geriatric assessment principles in T1D care, including routine screening for functional decline, fall risk, and multimorbidity. The development of integrated care models that address both medical and functional needs is essential. Future research should explore interventions targeting functional reserve and examine longitudinal trajectories of multimorbidity and functional status in T1D.

## Conclusion

In conclusion, this study demonstrates that multimorbidity is highly prevalent among Chinese adults with T1D and is strongly associated with older age and longer disease duration. Importantly, a higher burden of comorbidities is significantly linked to adverse functional outcomes, including increased fall risk and greater dependence in ADL, as well as substantially higher hospitalization costs. These findings highlight the critical need to integrate assessments of functional status, fall risk, and multimorbidity into routine clinical care for adults with T1D.

## Data Availability

The original contributions presented in the study are included in the article/**Supplementary Material**. Further inquiries can be directed to the corresponding author.

## Supplementary material

Supplementary Table 1 Associated factors for comorbidity in patients with T1D.

Supplementary Table 2 The prevalence and International Classification of Diseases (ICD) of sixty-one conditions among adult inpatients with T1D in the study.

Supplementary Figure 1 CONSORT diagram.

Supplementary Figure 2 Proportion of comorbid conditions among adults with type 1 diabetes, by different disease duration (<no><5</no> years, 5–10 years, ≥10 years) (top 20).

Supplementary Figure 3 Linear relationships between eight variables (ADL, fall risk, disease course, BMI, HbA1c, age, length of hospital stay, number of multimorbidities) and hospitalization costs. (372 patients were included in the cost analysis. Four patients were excluded due to pregnancy (n=1) and surgical treatment (n=3).
