# Supplementary material for: Multimorbidity and its associations with physical function, fall risk, and hospitalization cost in adults with type 1 diabetes: a cross-sectional study
Source: Front Endocrinol (Lausanne). 2026 May 7;17:1817819. doi: 10.3389/fendo.2026.1817819 (PMC13189871; doi:10.3389/fendo.2026.1817819)
Supplement: Supplementary Table 1 — Associated factors for comorbidity in patients with T1D. [file DataSheet1.docx]

Multimorbidity in type 1 diabetes is associated with decreased physical function and increased hospitalization cost: a cross-sectional study.

Supplement Table S1. Associated factors for comorbidity in patients with T1D.

Supplement Table S2. The prevalence and International Classification of Diseases (ICD) of sixty-one conditions among adult inpatients with T1D in the study.

Supplement Table S1. Associated factors for multimorbidities in patients with type 1 diabetes. * *P* <0.05

| Variables | OR (95%CI) | | | |
| --- | --- | --- | --- | --- |
|  | >=3 multimorbidities | >=4 multimorbidities | >=5 multimorbidities | >=6 multimorbidities |
| Gender，male vs female | 0.67 (0.41 ~ 1.12) | 0.92 (0.58 ~ 1.46) | 0.78 (0.47 ~ 1.30) | 0.98 (0.53 ~ 1.82) |
| BMI Normal vs Underweight | 0.75 (0.40 ~ 1.39) | 0.98 (0.53 ~ 1.78) | 1.15 (0.55 ~ 2.38) | 1.16 (0.46 ~ 2.95) |
| Overweight vs Underweight | 0.95 (0.45 ~ 2.02) | 1.68 (0.85 ~ 3.35) | 2.04 (0.92 ~ 4.50) | 1.71 (0.64 ~ 4.61) |
| HbA1c 6.5-9 vs <6.5 | 1.40 (0.65 ~ 3.04) | 1.29 (0.62 ~ 2.71) | 1.17 (0.50 ~ 2.76) | 0.81 (0.30 ~ 2.16) |
| HbA1c >=9 vs <6.5 | 1.12 (0.53 ~ 2.37) | 1.12 (0.54 ~ 2.32) | 1.06 (0.45 ~ 2.49) | 0.66 (0.25 ~ 1.77) |
| age 40-60 vs 18-39 | 4.45 (2.53 ~ 7.84) * | 3.18 (1.86 ~ 5.45) * | 2.26 (1.15 ~ 4.43) * | 2.95 (1.17 ~ 7.46) * |
| age >=60 vs 18-39 | 10.80 (4.92 ~ 23.70) * | 9.50 (5.09 ~ 17.74) * | 7.03 (3.59 ~ 13.76) * | 6.76 (2.75 ~ 16.61) * |
| Disease duration, years 5-10 vs 0-4 | 1.23 (0.63 ~ 2.38) | 1.71 (0.90 ~ 3.24) | 2.11 (1.01 ~ 4.41) * | 1.79 (0.70 ~ 4.55) |
| Disease duration, years >=10 vs 0-4 | 3.32 (1.77 ~ 6.22) * | 2.26 (1.33 ~ 3.84) * | 3.05 (1.69 ~ 5.51) * | 2.53 (1.21 ~ 5.26) * |

Abbreviations: BMI, body mass index; HbA1c, hemoglobin A1c

Supplement Table S2. The prevalence and International Classification of Diseases (ICD) of sixty-one conditions among adult inpatients with type 1 diabetes in the study.

| Comorbidities | Prevalence | ICD-10 |
| --- | --- | --- |
| Dyslipidemia | 0.511 | E78.000x001;E78.002;E78.100x006;E78.200;E78.200x003;E78.500;E78.600x006;R77.800x001 |
| Peripheral nerve disease | 0.314 | E10.400;E10.401+G63.2;E10.402+G99.0;E11.401+G63.2;E14.400x023+G63.2;G62.901 |
| Peripheral vascular disease | 0.303 | E10.500;E10.500x061+I79.2;E10.501+I79.2;E10.700x033;E14.500x011+I79.2;I25.101;I70.200x003;I70.203;I70.900x004;I74.300x030;I77.129 |
| Hypertension | 0.197 | I10.x00x002;I10.x00x023;I10.x00x024;I10.x00x027;I10.x00x028;I10.x00x032;I10.x03;I10.x04;I10.x05;I10.x09;I15.100x001 |
| Chronic kidney disease | 0.184 | E10.200;E10.200x211+N08.3;E10.200x212+N08.3;E10.200x213+N08.3;E10.200x215+N08.3;E10.201+N08.3;E14.200x210+N08.3;N18.802;N18.803;N18.804 |
| Cataract | 0.146 | E10.302+H28.0;E14.300x061+H28.0;E88.906+H28.1;H25.900;H26.200;H26.400;H26.801;H26.900;H28.0;Z96.100x001;Z96.101 |
| Anaemia | 0.109 | D50.801;D50.900;D56.000;D56.100;D56.900;D64.900;D64.901;D64.902;N18.800x017;O99.006 |
| Cancer | 0.09 | C00-97; M80700/3;M81470/3;M83120/3;M95910/3;Z51.100x002;Z85.101;Z85.300;Z85.407;Z85.501;Z85.804;Z85.809;Z98.800x502 |
| Nonalcoholic fatty liver disease | 0.085 | K70.000;K76.000;K76.000x011 |
| Hashimoto thyroiditis | 0.077 | E06.304 |
| Hyperthyroidism | 0.059 | E05.000x001;E05.000x002+H06.2*;E05.800x001;E05.805;E05.900;E05.900x001 |
| Hepatitis B virus | 0.059 | B16.904;B18.101;B18.107;K74.600x003;Z22.500x002;Z22.502 |
| Coronary heart disease | 0.048 | I24.800x007;I25.102;I25.103;I25.901 |
| Hypothyroidsim | 0.043 | E03.201;E03.800x001;E03.800x002;E03.900 |
| Chronic obstructive pulmonary disease | 0.037 | J43.900;J43.900x011;J44.900 |
| Osteoporosis | 0.035 | M81.400;M81.800x091;M81.900;S22.000x003 |
| Cerebrovascular disease | 0.027 | I63.801;I63.900;I69.300;I69.300x002 |
| Depression | 0.024 | F32.900;F32.901;F38.001;F41.200x002;F53.002 |
| Anxiety | 0.024 | F41.101;F41.102;F41.200x002 |
| Hearing disorders | 0.019 | H90.100;H90.300;H90.400;H90.500;H91.900;H93.200 |
| Macular degeneration | 0.019 | H35.300x001;H35.305;H35.804 |
| Malnutrition | 0.016 | E43.x00x003;E44.000x001;E46.x00;E46.x00x003 |
| Glaucoma | 0.013 | H40.203;H40.403;H40.900;Z54.000x033 |
| Bronchiectasis | 0.013 | J47.x00;J47.x03 |
| Heart failure | 0.013 | I50.900x002;I50.900x018;I50.907 |
| Osteoarthritis | 0.011 | M17.000;M17.900;M17.900x002 |
| Eczema | 0.011 | L29.801;L30.902;L30.904 |
| Peptic ulcer | 0.011 | K25.900x001;K26.701 |
| Polycystic ovary syndrome | 0.008 | E28.200 |
| Rheumatoid arthritis | 0.008 | M06.900 |
| Insomnia | 0.008 | G47.000x001;G47.900 |
| Reflux esophagitis | 0.008 | K21.001 |
| Intersititial pneumonia | 0.008 | J84.900x002 |
| Epilepsy | 0.008 | G40.600;G40.800x004;G40.900 |
| Hypofunction of the anterior pituitary gland | 0.005 | E23.000x008 |
| Chronic urticaria | 0.005 | L50.802 |
| Obstructive sleep apnea syndrome | 0.005 | G47.300x001;G47.300x037 |
| Pulmonary hypertension | 0.005 | I27.200x012;I27.200x015 |
| Atrial fibrillation | 0.005 | I48.x00x021 |
| ANCA associated vasculitis | 0.003 | M31.802 |
| Gout | 0.003 | M10.900 |
| Polymyositis | 0.003 | M33.200 |
| Uveitis | 0.003 | H20.900x004 |
| Hypoparathyroidism | 0.003 | E03.201;E03.800x001;E03.800x002;E03.900 |
| Adrenocortical hypofunction | 0.003 | E27.300;E27.407 |
| Schizophrenia | 0.003 | F20.900 |
| Vitiligo | 0.003 | L80.x00 |
| Liver failure | 0.003 | K72.001 |
| Hepatitis C virus | 0.003 | B18.200 |
| Inflammatory bowel disease | 0.003 | K50.900 |
| Asthma | 0.003 | J45.900x022 |
| Parkinson's disease | 0.003 | G20.x00 |
| Guillain-barre syndrome | 0.003 | G61.000 |
| Rickets | 0 | E55.001 |
| Femoral head necrosis | 0 | M87.002 |
| Sjogren's syndrome | 0 | M35.000 |
| Systemic sclerosis | 0 | M34.900x001;M34.900 |
| Rheumatic arthritis | 0 | M06.900 |
| Syndrome of inappropriate secretion of antidiuretic hormone | 0 | E22.200x001 |
| Psoriasis | 0 | L40.900 |
| Atrophic Gastritis | 0 | K29.400x001 |
