# Supplementary figures and images for: Multimorbidity and its associations with physical function, fall risk, and hospitalization cost in adults with type 1 diabetes: a cross-sectional study
Source: Front Endocrinol (Lausanne). 2026 May 7;17:1817819. doi: 10.3389/fendo.2026.1817819 (PMC13189871; doi:10.3389/fendo.2026.1817819)

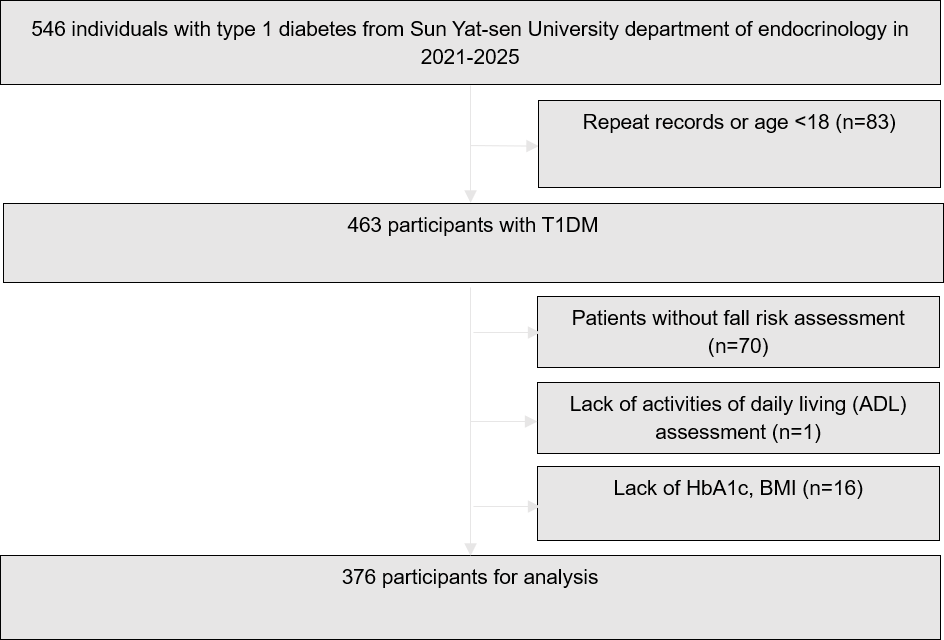

Supplement: Supplementary Figure 1 — CONSORT diagram. [file Image1.tif]

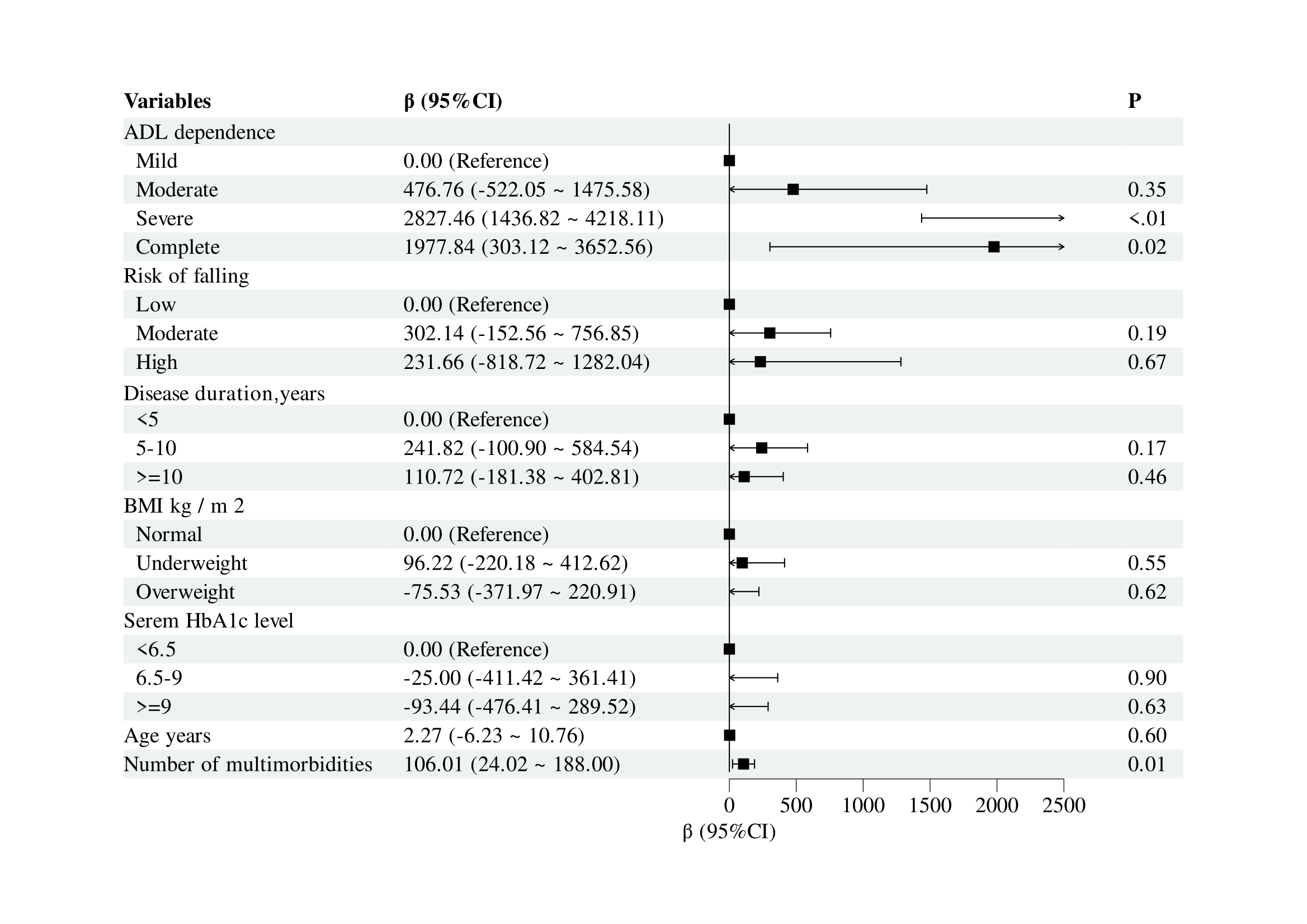

Supplement: Supplementary Figure 3 — Linear relationships between eight variables (ADL, fall risk, disease course, BMI, HbA1c, age, length of hospital stay, number of multimorbidities) and hospitalization costs. (372 patients were included in the cost analysis. Four patients were excluded due to pregnancy (n=1) and surgical treatment (n=3). [file Image3.tiff]

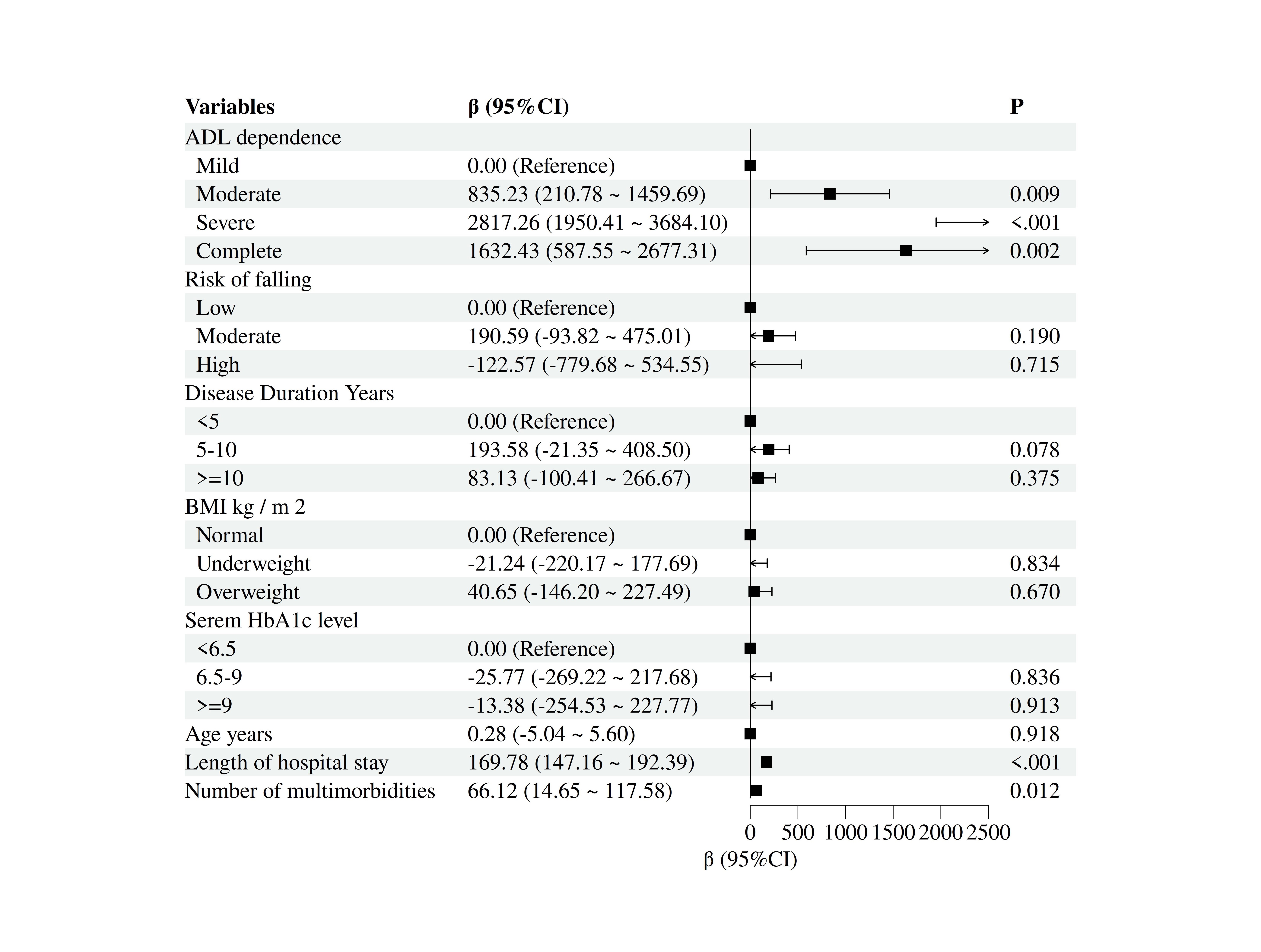

Supplement: Supplementary file 5 [file Image4.jpeg]
